# Enhancing the design of wine labels

**DOI:** 10.3389/fpsyg.2023.1176794

**Published:** 2023-09-25

**Authors:** Anders Crichton-Fock, Charles Spence, Maria Mora, Nicklas Pettersson

**Affiliations:** ^1^School of Hospitality, Culinary Arts & Meal Science, Örebro University, Örebro, Sweden; ^2^Crossmodal Research Laboratory, Department of Experimental Psychology, Oxford University, Oxford, United Kingdom; ^3^BCC Innovation, Technology Center in Gastronomy, Basque Culinary Center, Donostia-San Sebastián, Spain; ^4^Basque Culinary Center, Faculty of Gastronomic Sciences, Mondragon Unibersitatea, Donostia-San Sebastián, Spain; ^5^School of Business, Örebro University, Örebro, Sweden

**Keywords:** crossmodal communication, correspondence, multisensory, vision, wine assessment, complex foods, resource efficiency, wine labels

## Abstract

**Introduction:**

The knowledge accrued through research in the domain of crossmodal correspondences has had a significant influence on a diverse array of disciplines, including behavioral studies, neuroscience, computational modeling, and notably, marketing, with the objective of aligning sensory experiences to help shape patterns of consumer behavior. A study is reported that explores the extension of these principles to the communication of products having a notably complex sensory profile, specifically within the context of wine. The central aim of the project is to explore the feasibility of using crossmodal communication as a strategic tool to augment the congruence between the consumers’ multisensory expectations and their sensory experiences. For consumers venturing into the realm of wine selection without the advantage of prior tasting experience, it is of paramount importance to possess a robust understanding of the mandated information. This encompasses critical elements such as the wine’s origin, grape varietal(s) used, geographical indications, producer qualifications, and the potential implications of these factors on the final wine product. This level of comprehension stands as a necessary prerequisite, enabling these consumers to make informed choices that align with their preferences, even in the absence of previous sensory encounters. Nonetheless, semiotic investigations underscore the significance attributed to symbolic components such as signs, logos, colors, gestures, and linguistic cues. Research from the field performing multisensory studies, presents a counterpoint to prevailing communication paradigms, advocating for a heightened incorporation of metaphors, analogies, symbols, metonymies, and allegories. This alternative approach aims to enhance the efficacy of communication strategies, offering a more profound and evocative means of conveying intricate messages on a more holistic level.

**Methods:**

A questionnaire was sent to a specific group of engaged wine consumers (*n* = 329). Besides questions regarding demographics, purchase behavior, and consumption behavior, the questionnaire included examples of multisensory communication through a selection of symbols, as well as alternative wine information.

**Results:**

The results showed significant correlations between demographics, consumption behavior, and attitudes toward the tested multisensory symbols and alternative information, thus helping to gain a better understanding of the sensory properties that should be communicated on wine labels.

**Discussion:**

The findings reported here highlight the effectiveness of visual crossmodal communication as a promising pathway capable of skillfully capturing consumer attributes, conveying multisensory experiences, and portraying the comprehensive timeline of taste evolution. As a result, it assumes a pivotal role as a communicative tool for intricate consumables, like wine, functioning at the crossroads of visual and sensory dimensions.

## 1. Introduction

In the realm of sensory marketing, the utilization of crossmodal correspondences has emerged as a strategic approach to harness the manifold multisensory effects elucidated through an expanding array of controlled laboratory investigations. This primary strategy involves leveraging insights into the intricacies of consumer behavior and contentment, thereby serving as a mechanism to augment sales and fortify market standing within the competitive market environment (Goldkuhl and Styvén, 2007; Krishna, 2010; Krishna and Schwarz, 2014). While research on the crossmodal correspondences that has been published to date has primarily focused on understanding cognitive stimulation and intermodal connections, the application in sensory marketing has targeted various sensory experiences that might benefit from it. By collectively influencing the manner in which consumers perceive and engage with products, particularly in the context of communication and alignment with consumers’ expectations of the products (Weil, 2007). Vision is widely considered the dominant sense to use in this context (Hutmacher, 2019).

In essence, sensory marketing seeks to investigate how sensory cues influence the consumer’s encounters from a commercial perspective (Wang and Li, 2022). Notably, within this field, vision tends to take precedence. Consequently, it becomes essential to establish a seamless alignment between consumer preferences and the attributes that a potential product can offer and by so doing create a more harmonious multisensory experience (Elder and Krishna, 2010; Varela and Ares, 2012; Paradis and Eeg-Olofsson, 2013; Krishna and Schwarz, 2014; Croijmans and Wang, 2021).

Consumer research includes many possible approaches and multiple cultural and genetic factors to consider (Bartoshuk et al., 1996; Bartoshuk, 2000; Reed and Knaapila, 2010; Pagliarini et al., 2021). For instance, at the sensory level, researchers have explored the genetic impact of taste sensations in regard to consumers’ perceptual sensitivity to, and preference for, certain key attributes, such as sweetness (Gent and Bartoshuk, 1983), sourness (Breslin, 1996; Pagliarini et al., 2021), bitterness (Bartoshuk et al., 1988), saltiness (Breslin and Beauchamp, 1997; Bartoshuk et al., 1998), and umami (Keast and Breslin, 2003; Kim et al., 2015; Linscott and Lim, 2016). Beyond crossmodal interactions, integrating genetic factors becomes pertinent in the pursuit of enhancing communication by targeting pivotal sensory attributes that influence consumer perception and acceptance of specific food products amongst particular groups of consumers. This becomes particularly relevant when examining the divergent reactions of various groups of consumers to a given product, even though genetic research might exhibit certain limitations in pinpointing such responses. This challenge is notably intricate when addressing multifaceted flavor profiles and the aromas of certain food products, with wine serving as a prime example of such stimulus complexity (Shepherd, 2006; Parr, 2015; Spence and Wang, 2018). Adding to the communication challenge, the production of wine involves multiple stages of refinement, spanning from cultivation to bottling which, in turn, contributes to a notable climate impact as this intricate process unfolds (Christ and Burritt, 2013; Iannone et al., 2016).

In the domain of packaging, despite the transient ebb and flow of diverse trends involving motifs such as critters and idiomatic expressions, wines continue to be characterized by labels that can be classed as conventional. These labels, primarily affixed to the front and back of wine bottles, predominantly serve as conduits for obligatory and regulated content, as dictated by prevailing legislative frameworks. The conventional labeling conventionally encompasses details pertaining to the wine’s provenance, country of origin, grape varietal, alcohol concentration, and vintage year. However, it is worth noting that these traditional designs may not inherently convey the intricate nuances of a wine’s sensory properties to the discerning consumer. In light of the genetic influences and crossmodal factors elucidated by prior research, it becomes relevant to explore the feasibility of incorporating these conceptual frameworks into the design of wine labels in order to investigate their potential use in consumer communication.

The present study aimed to investigate consumer attitudes toward crossmodal and multisensory approaches to wine communication, using both visual and non-verbal cues to help communicate various multisensory information on the label. The second aim was to investigate critical attributes and other information requested by consumers in order to examine the paradigm of conventional wine labeling. More effective communication can thus better cater to specific target groups while optimizing the use of natural resources in terms of satisfying the consumer.

### 1.1. Literature review

Studies in semiotics, exploring symbolic communication and understanding, propose that various forms of meaning such as signs, logos, gestures, illustrations, linguistic and non-linguistic communication can serve as essential tools when it comes to engaging different groups of consumers (König and Lick, 2014; van Tonder and Mulder, 2015; Lick et al., 2017; Celhay and Remaud, 2018; Pelet et al., 2020). Beyond semiotics, researchers have also advocated for the use of rhetorical figures such as metaphors, analogies, symbols, metonymies, and allegories to enhance communication effectiveness (Moreno Lara, 2014; Alousque, 2015; Kelley et al., 2015; Costello et al., 2018; Herdenstam et al., 2020). Furthermore, exploration into modern and innovative communication strategies has considered sensory descriptors to evoke olfactory mental imagery (Shepherd, 2006; Tomiczek and Stevenson, 2009), as well as the use of scene or country descriptions or origin to help construct a mental sensory experience to promote sale (Tomiczek and Stevenson, 2009; Williamson et al., 2016; Croijmans and Wang, 2021).

Furthermore, researchers have ventured into novel sensory strategies, including the integration of multisensory or crossmodal stimuli, in order to align consumer expectations with the tasting experience. This involves communicating the impact of specific food combinations (Harrington, 2005, 2008; Koone et al., 2014; Herdenstam et al., 2018; Spence, 2020b) and identifying attributes in these combinations that might impact consumer acceptance (Harrington, 2005, 2007; Harrington and Hammond, 2006, 2007, 2009; Harrington et al., 2010; Koone et al., 2014; Harrington and Seo, 2015).

In the realm of crossmodal correspondences, associations between the stimuli presented (or merely imagined) in one sensory modality affecting responses in another modality have been studied (Spence, 2011). Notably, within sensory analysis, crossmodal interactions have demonstrated varied impacts on consumer perceptions within different dining and food contexts. Research in this domain has explored influences ranging from frequency of sound and music (Spence et al., 2014; Hagtvedt and Brasel, 2016; De Luca et al., 2019), lighting and colors (Spence et al., 2014; Biswas et al., 2017; Heatherly et al., 2019; Maziriri et al., 2021), visually-presented shapes (Hanson-Vaux et al., 2013; Liu et al., 2018; Spence, 2020a,b), touch and tactile sensations (Gallace and Spence, 2010; Spence et al., 2013; Etzi et al., 2014; Olzak and Craig, 2014; Wang and Spence, 2018a,b), and, not least, the significant influence of odors and scents (Goldkuhl and Styvén, 2007; Krishna, 2010; Crisinel and Spence, 2012; Deroy et al., 2013; Reid et al., 2015; Ward et al., 2022; Spence, 2022b).

Furthermore, other researchers have explored contemporary and innovative methods for communicating essential and desirable product attributes. These approaches include using sensory descriptors to help conjure up olfactory mental imagery (González et al., 2006; Shepherd, 2006; Tomiczek and Stevenson, 2009), as well as investigating the effects of describing scenes or countries to craft a detailed mental image of a particular sensory encounter (Tomiczek and Stevenson, 2009; Williamson et al., 2016; Croijmans and Wang, 2021).

In summary, the phenomenon of crossmodal correspondence has been extensively studied in various consumer contexts, revealing its substantial influence on consumer satisfaction. However, to the best of the authors’ knowledge, there appears to be a research gap regarding the potential application of crossmodal communication to enhance the consumer’s comprehension of the expected intricate sensory attributes in a complex tasting experience, such as offered by a quality wine. These products, characterized by layers of volatile odors, flavors, and oral-somatosensory sensations on a multisensory level, could hold critical importance for achieving consumer approval (Wang and Spence, 2018a,b). If effectively harnessed to cater to specific target audiences, crossmodal communication might thus not only help to bolster marketing strategies and consumer contentment but also serve as a tool for optimizing the use of resources within complex food products, potentially contributing to the broader goal of reducing food waste (Galbreath et al., 2020).

## 2. Materials and methods

### 2.1. Ethics statement

The questionnaire was performed in accordance with the Declaration of Helsinki and the European Code of Conduct for Research Integrity. All of the respondents were over 20 years of age, and informed consent was obtained from all respondents. All data and analysis files were kept in accordance with legislated and regulated data handling practices.

### 2.2. Respondents

The sample consisted of 329 students from different sections of the 7.5-credits, 15-week distance course ‘Beverage knowledge’ offered at Örebro University (Sweden). These students underwent comprehensive training and gained collective proficiency in wine analysis, along with experience in crossmodal correspondence. This experience was particularly evident during training and tasting sessions, wherein respondents engaged in meticulous evaluations while transitioning between senses, leading to significant preconceived notions. The assessment process involved a sequence, starting with visual evaluations encompassing aspects like color, intensity, maturity, age, freshness, acidity, and concentration. These preliminary impressions were subsequently corroborated through olfactory assessments and later confirmed on the palate. This approach provided respondents with firsthand encounters of crossmodal influences and their noteworthy impact during professional wine tasting procedures. This impact was exemplified when respondents engaged in diverse tasting exercises. For instance, they initially perceived fragrance notes of ripe pineapple and sweet mango, thus forming initial impressions concerning the wine’s perceived level of sweetness. However, upon tasting, they realized that the wine was, in fact, completely dry. This experiential interplay of senses distinctly highlighted the intricate interrelationship between sensory modalities and their potential to substantially influence the overall perceptual experience.

The majority of the respondents were female (59%) and lived in the city (76%). Almost all had previously studied at the university (94%), and most of them had received a bachelor’s degree or higher (74%). Many considered themselves to have better wine knowledge than the population at large (76%). Most of them consumed wine on a weekly base (87%), which they typically purchased at Systembolaget (Sweden’s nationally regulated liquor monopoly) (81%) and consumed at home (80%) (see Supplementary Appendix A).

The respondents shared the following traits:

- They had all tasted the same wines and other beverages, and therefore shared a variety of sensory experiences (see Supplementary Appendix F).
- They had all learned a common approach and methodology for analyzing wine. It can thereby be presumed that, on a group level, they had an awareness of the importance of all sensory modalities in the analysis process, including vision, smell, taste, touch, and sound.
- They had all been exposed to crossmodal correspondence during the course. This by performing the large number of tasting exercises involved in the course. Especially when moving from one sense to another during the tasting process, and, subsequently, communicating it, while each sensory modality is not separately (see Supplementary Appendix F).

### 2.3. Questionnaire

The questionnaire consisted of four sections. The first section included questions relating to *demographics*, as well as single-choice and multiple-choice (check-all-that-apply; CATA) questions relating to purchase and consumption behaviors, communication, and sensory experiences (see Supplementary Appendix B). The second section of the questionnaire aimed to test different *design and symbols* developed in dialogue with wine experts and researchers. The symbols and illustrations used in this section attempted to assess the perception of several of the multisensory factors that have been shown to affect crossmodal experiences. The illustrations were developed by the art and food designer Elin Aronsen Beis, who also specializes in food packaging. For each design question, a short background was given to briefly illustrate the communicative purpose of each symbol, item, or other piece of information. After exposure to the different designs, the respondents were asked to indicate on a 7-point Likert scale (1 = “Not very helpful”; 4 = “Neutral”; 7 = “Very helpful”) how helpful each design was in interpreting the potential sensory characteristics of the product.

**Figure 1:**
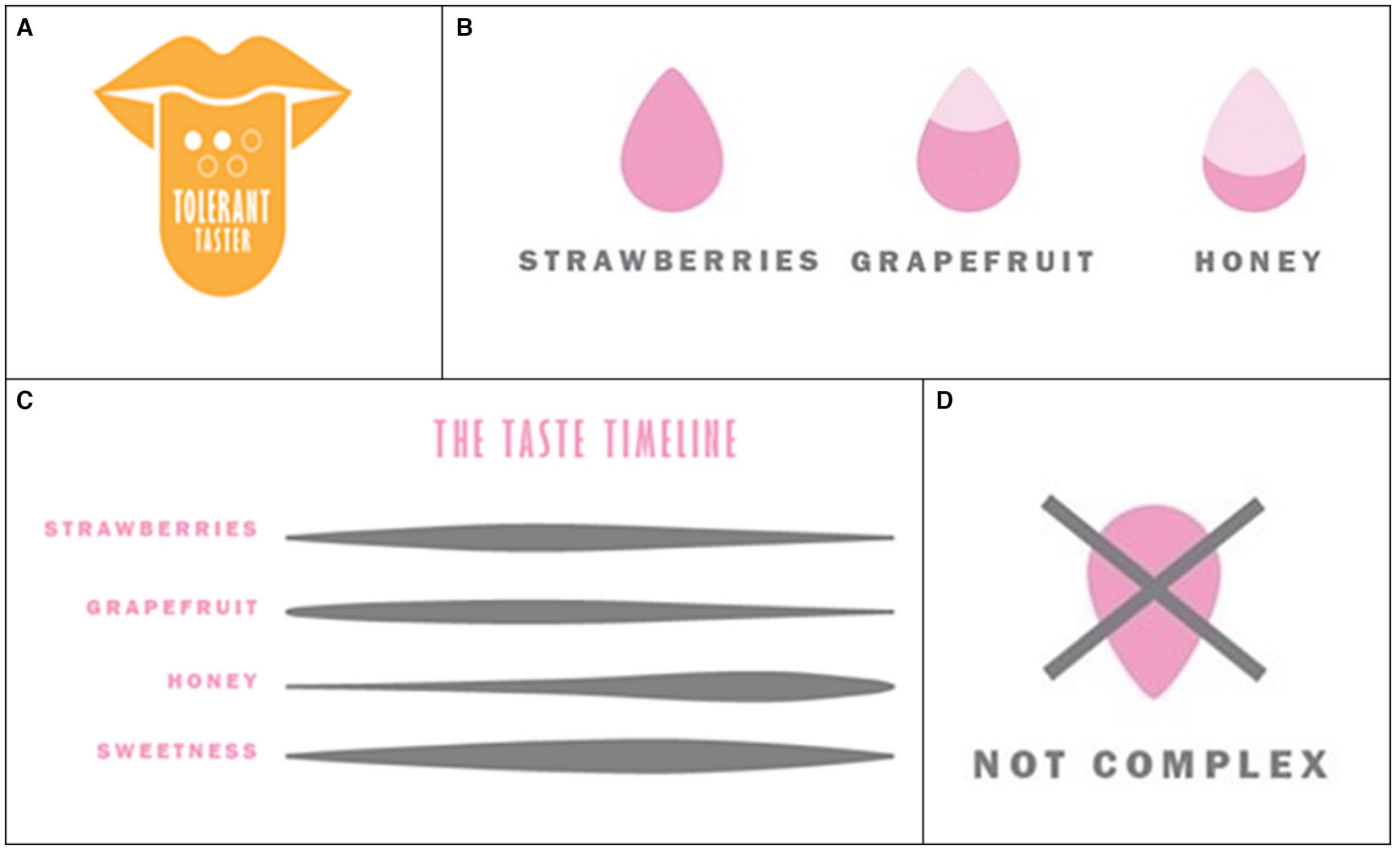
Symbol in the questionnaire used to illustrate an alternative communication approach based on; **(A)** genetics and sensitivity; **(B)** dominant flavors in the wine; **(C)** the temporal dynamic change during the tasting experience – from when the wine hits the nose and mouth (attack), development on the palate (mid-palate), and the duration of flavors in the end (finish); **(D)** of non-existent qualities in the wine – such as the lack of complexity.

Here follows an example of a creative design question included the questionnaire and respective background information given to the respondents before answering each question:

*On the group level, the research shows that consumers have different sensitivities to bitterness that affect our preference for wine depending on consumers’ taste type (tolerant, sensitive, very sensitive, and hyper-sensitive). To what extent do you think it would be helpful to communicate the optimal consumer taste group on the label (see example)?* (see Figure 1A). The idea being both to present research in this field as well as how it could be implemented in consumer communication. One aspect being that the consumer already has awareness of their own “optimal consumer taste group,” another whether this information would be helpful if added to a wine label. The other design questions included other symbols and illustrations (see Figures 1B–D) as well as background information to stimulate the creative process and understand the context of use for each symbol.

**Figure 2:**
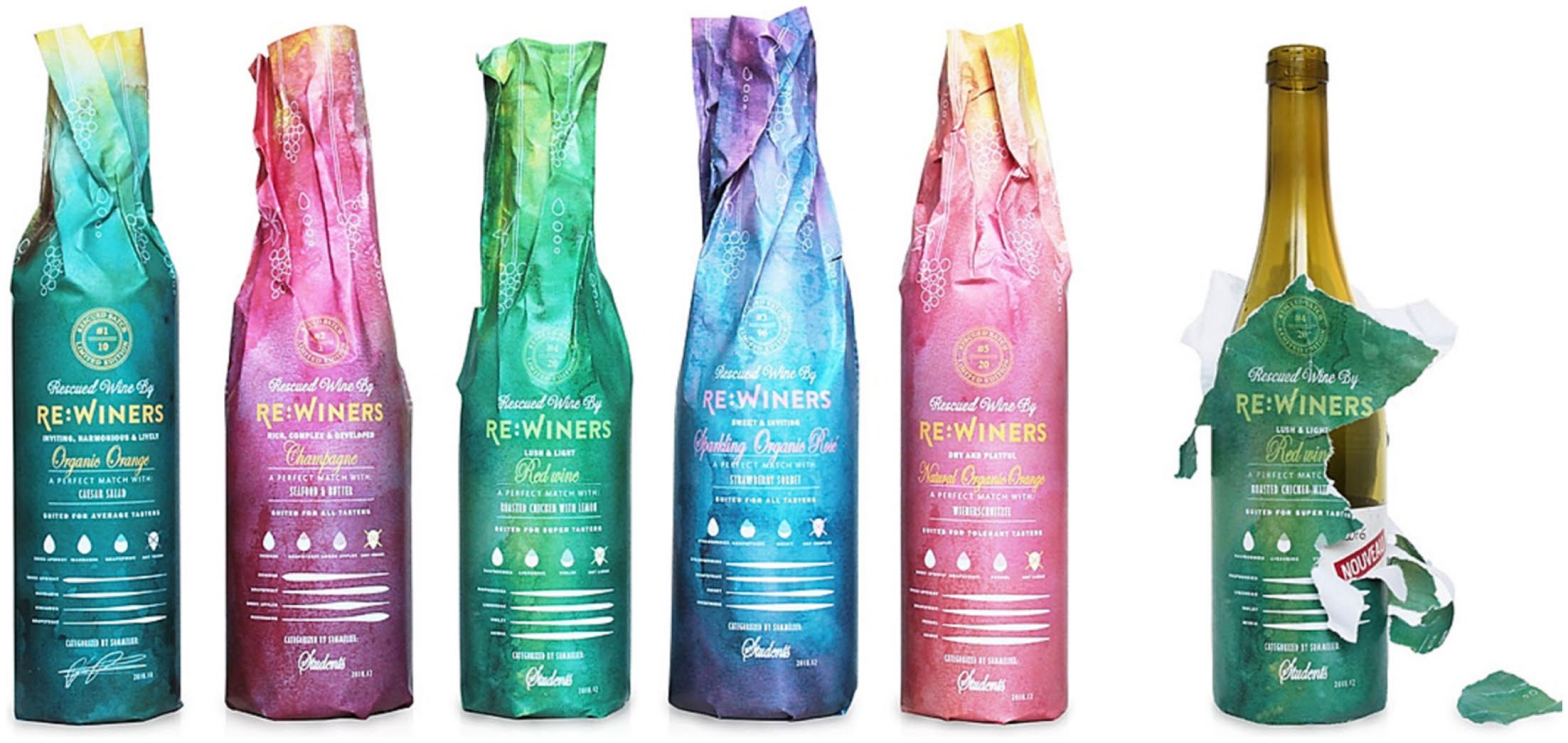
An image that was included in the questionnaire, intended to illustrate an innovative method of label communication. This approach involves using relabeled wine bottles featuring solely sensory descriptors. The design of this concept was created by Elin Aronsen Beis.

In the third section, the respondents were asked about their preferred *textual sensory descriptions and assessments* to be included in label of wines of different origins (see Figure 2).

Within this segment, various other inquiries concerning labels were also presented. These included evaluations conducted by experts, encompassing factors like the readiness of the wine for consumption and judgments on quality. Moreover, the third section of the questionnaire directed respondents’ attention toward the potential inclusion of insights from professional tasters. This section aimed to gauge whether communicating common faults and defects typically associated with a specific type of wine would be beneficial. The underlying rationale behind these inquiries shifted from the prior questions, which had focused on more personalized engagement and self-awareness. For instance, respondents were asked about their awareness of their “optimal consumer taste group.” The intention behind incorporating these queries involving wine experts, as opposed to the previous questions concerning personal knowledge, was to facilitate a comparison between diverse communication strategies. This comparison aimed to shed light on the efficacy of different approaches in conveying information to consumers. In the fourth and final section, the respondents were asked questions related to alternative sensory communication in general as well as attitudes toward buying blended wines, wines made from already existing wines, and sustainability.

### 2.4. Data analysis

EyeQuestion version 5 (Logic 8, Elst, The Netherlands), a software program for sensory and consumer testing, was used to collect the respondents’ responses. Statistical analysis was undertaken using the software R (R Core Team, 2021).

## 3. Results

### 3.1. Attitudes toward tested wine label design and symbols (questionnaire section 2)

Regarding attitudes toward alternative communication using the tested symbols, the respondents showed positive responses (above neutral) toward symbols illustrating dominant *flavor intensity* (76%) and *taste timeline/flavor development* (65%). Symbols for *non-existing qualities* (52%) and *genetics and taste sensitivity* profiling (49%) received a positive response from approximately half of the respondents (see Table 1). For more details, see Supplementary Appendix D.

**Table 1:** Attitudes toward tested symbols.

Frequency (*n* = 329)	Genetics (%)	Flavor intensity (%)	Taste timeline (%)	Non-existing qualities (%)*
7. Very helpful	6.6	18.0	14.2	11.4
6.	9.8	29.3	20.2	17.0
5.	32.2	29.0	30.9	23.7
4. Neutral	32.2	13.3	20.2	21.5
3.	7.6	6.6	7.3	7.9
2.	6.6	1.9	3.8	8.8
1. Not very helpful	5.1	1.9	3.5	9.8

* Communication of absent sensory qualities that might affect acceptance on individual level.

### 3.2. Attitudes toward tested textual sensory descriptions and assessments by wine experts (questionnaire section 3)

For the tested text information, respondents showed a positive response toward *level of readiness* (89%) and *quality assessment* by a wine expert (71%). Just over half of respondents (53%) responded positively toward highlighting *potential faults* (see Table 2).

**Table 2:** Attitudes toward other assessments/scale.

Frequency (n = 329)	Quality assessed by professional wine expert/ Poor – Outstanding. (%)	Readiness (to drink) assessed by professional wine expert/ Too young – Too old. (%)	High-risk faults assessed by professional wine expert/ Frequent wine faults. (%)
7. Very helpful	19.2	40.1	12.0
6.	28.4	27.4	18.3
5.	23.0	21.5	23.0
4. Neutral	15.1	6.6	23.7
3.	6.0	1.9	11.7
2.	5.0	0.3	5.0
1. Not very helpful	3.2	2.2	6.3

Only text and no symbol presented.

### 3.3. Attitudes toward alternative sensory communication, blending, and sustainability (questionnaire section 4)

A majority of the respondents (64%) said that they would be open to at least try a bottle based on sensory information alone, while approximately 9% answered that they would never consider it. As for buying wine that had been made through a blend of other existing wines, most respondents (74%) reported that they would try a bottle, while about 5% reported that they would never consider it. Regarding attitudes toward sustainability, a majority of the respondents (76%) reported that they take this into consideration at least to some degree when purchasing wine. By contrast, about 6% of respondents answered that they would never take this into account when buying wine (see Table 3). For more information, see Supplementary Appendix D.

**Table 3:** Other attitudes regarding sensory labeling, blending wines and sustainability.

Choice (*n* = 329)	Sensory information (%)	Blending (%)	Choice (*n* = 329)	Sustainability (%)
This would suit me	1.6	3.5	Always	2.2
This sounds like something that I would like	15.9	14.9	Most of the time	22.9
I would be willing to try a bottle	46.0	55.2	Sometimes	50.8
I might if the descriptors suited my palate	27.9	21.0	Rarely	18.4
Never	8.6	5.4	Never	5.7

### 3.4. Bivariate analysis of consumer attitudes toward alternative wine label communication related to reported demographics and purchase and consumption behaviors

The bivariate relationships between attitudes, demographics, and behaviors were analyzed *via* the Kendall’s Tau rank correlation coefficient, except for the non-binary nominal demographical factors, where the Kruskal–Wallis test of equality of average rank among groups was used.

#### 3.4.1. Results – associations between demographics and the perceived helpfulness of alternative label communication

As shown in Table 4 and Supplementary Appendix D, no significant associations were found between any of the demographic traits and the helpfulness of certain communication—neither the optimal consumer taste group based on genetics, nor the taste timeline in order to match a certain preference, nor the intensity of the dominating flavors. Likewise, the helpfulness of alternative label communication was not significantly associated with where the respondents happened to live, their knowledge about wine, where they primarily consume their wine, or the degree to which they select wine based on sustainability (decreasing the climate footprint). There were, however, certain significant associations:

- The helpfulness of being informed of properties that the wines do not have was positively correlated with how often respondents consume wine.
- The helpfulness of professionals to communicate the quality was negatively correlated with being born outside of Sweden and positively correlated with age, the level of highest completed education, and being a Swedish resident.
- The helpfulness of a professional assessment of the consumption readiness of the wine was positively correlated with being female, the level of highest completed education, and the level of income.
- The helpfulness of communicating potential faults was positively correlated with age (q2) and where respondents primarily purchased wine.
  For more details, see Supplementary Appendix D.

**Table 4:** Correlations (Kendall’s tau) between demographics and perceived helpfulness of alternative label communication.

	Perceived helpfulness of alternative label communication						
Demographical variable	Genetics	Flavor intensity	Taste timeline	Non-existing qualities (x)	Quality by pro	Readiness (to drink)	High-risk faults
Age	0.029	−0.012	−0.004	0.038	0.083 *	0.080	0.144 ***
Highest completed education	−0.027	−0.013	−0.003	0.083	0.112 *	0.131 **	0.068
How often do you consume wine?	0.025	−0.004	0.026	0.105 *	0.050	0.068	0.074
How much do you know about wine?	−0.065	−0.062	−0.018	0.008	0.008	−0.037	−0.035
What is your monthly income? (Before taxes.)	−0.014	−0.033	0.01	0.078	0.078	0.132 **	0.048
To what degree do you select wine in regard to sustainability - decreasing the climate footprint?	0.061	0.040	0.012	0.001	−0.026	0.001	−0.061

Two-tailed value of *p*: 0.05 > * > 0.01 > ** > 0.001 > ***. (x) Communication of absent sensory qualities that might affect acceptance on individual level.

#### 3.4.2. Results – correlations between purchasing behavior and perceived helpfulness of alternative label communication

As shown in Table 4, no signification correlations were found between the helpfulness of being informed about properties that the wines did not have and purchasing behavior. Similarly, the helpfulness of alternative label communication was not significantly correlated with the factors influencing the choice of wine such as price, grape, country of origin, climate impact, style, vintage, front label, back label, and label illustrations. The following significant correlations were found with factors influencing purchasing behavior:

- The helpfulness of the optimal consumer taste group based on genetics was negatively correlated with previous experience.
- The helpfulness of the taste timeline in order to match a certain preference was positively correlated with sensory indicators.
- The helpfulness of the intensity of the dominating flavors was positively correlated with sensory indicators and external recommendations.
- The helpfulness of professionals to communicate the quality was negatively correlated with the wine producer and bottle design.
- The helpfulness of professional assessment of wines readiness was positively correlated with external recommendations.
- The helpfulness of communicating potential faults was positively correlated with previous experience.

#### 3.4.3. Results – correlations between perceived helpfulness of alternative label communication and consumption behavior

Regarding the question concerning influencing factors during the consumption of wine and the sensory experience, no significant correlations could be found between influencing factors and a preference for certain label communication. One possible reason for this might be the complexity of the question, which presupposes an understanding of the influencing factors. The lack of understanding of these influencing factors and their impact in the consumption context may be potential areas of further exploration to improve consumer communication (see Supplementary Appendix E).

### 3.5. Regression analysis of perceived helpfulness of alternative wine label communication in relation to reported demographics, purchase behaviors, and consumption behaviors

To compare the rating of the various attitudes toward the helpfulness of different types of alternative communication as well as their relationship to demographics, purchase behaviors, and consumption behaviors, a cumulative linked mixed model was fitted. The within-participant rating correlation was modeled by participant random intercepts. First, the ratings were regressed on the; variable categories; demographics; purchase behaviors; and consumption behaviors. Then, the non-significant variables were eliminated until all the remaining estimated effects significantly differed from zero, see Table 5.

**Table 5:** Correlations (Kendall’s tau) between purchasing behavior and perceived helpfulness of alternative label communication.

	Perceived helpfulness of alternative label communication						
When purchasing wine from your selected choice in the above question, what factors influence your choice?	Genetics	Flavor intensity	Taste timeline	Non-existing qualities (x)	Quality by pro	Readiness (to drink)	High-risk faults
The country of origin	−0.023	−0.003	−0.03	0.020	0.029	0.015	−0.003
The grape	0.026	0.051	−0.013	0.085	0.034	0.037	0.041
The style	−0.003	0.039	0.030	0.022	0.060	−0.001	−0.017
Illustrations on the label	−0.012	0.065	−0.028	0.059	−0.020	−0.033	−0.030
The wine producer	−0.006	−0.008	0.014	0.068	−0.133 **	0.053	0.040
Sensory indicators	0.039	0.153 **	0.103 *	0.037	0.038	0.078	0.095
External recommendations	−0.075	−0.051	0.121 *	0.028	0.004	0.120 *	0.017
Previous experience	−0.102 *	−0.080	0.000	0.018	0.045	−0.012	0.144 **
Climate impact	−0.014	0.041	0.002	−0.031	0.005	0.049	−0.059
Price	−0.020	0.029	0.009	−0.010	−0.056	−0.026	−0.027
Bottle design	−0.069	−0.036	−0.023	0.034	−0.117 *	−0.014	−0.055
Front label	−0.032	0.029	0.002	0.079	−0.07	−0.015	−0.064
Back label	−0.085	−0.050	−0.042	0.082	0.002	−0.087	−0.096
Vintage	−0.025	−0.094	−0.065	0.037	0.029	−0.003	0.035

Two-sided value of *p*: 0.05 > * > 0.01 > ** > 0.001 > ***. (x) Communication of absent sensory qualities in the product that might affect acceptance on individual level.

The magnitude of the helpfulness questions (value of *p* = 0.000) resembled the univariate results in Sections 3.1 and 3.2, where the level of readiness had the highest and genetics and taste sensitivity profiling and non-existing qualities had the lowest helpfulness rating, while the other categories fell in between. The demographical and behavioral variables found to be significant in the regression provide indications of general patterns among attitudes toward the helpfulness of alternative wine label communication. All of them also had some significant bivariate relationships to the helpfulness questions (see Section 3.4). Being older (value of *p* = 0.014) and being female (value of *p* = 0.035) both had positive estimated effects, corresponding to higher expected helpfulness ratings. Furthermore, wine purchasing behavior influenced by sensory indicators had a positive effect (value of *p* = 0.009) while primarily consuming wine in restaurants and bars (value of *p* = 0.005) had a negative effect (Table 6).

**Table 6:** Significant effects from the regression of attitudinal ratings toward the perceived helpfulness of alternative communication on demographical, consumption and purchasing behavioral variables (cumulative linked mixed model).

	Perceived helpfulness of alternative label communication variable						
Category	Genetics	Flavor intensity	Taste timeline	Non-existing qualities (x)	Quality by pro	Readiness (to drink)	High-risk faults
Estimated effect (standard error)	0	0.83 (0.14) ***	1.33 (0.15) ***	0.13 (0.14)	1.18 (0.15) ***	2.46 (0.16) ***	0.37 (0.14) **

	Demographic, consumption and purchasing behavioral variables						
Variable and category	Age	Gender		Consume in		Influential on choice	
	10-year effect	Male	Female	Other	Bar or restaurant	Sensory indicator No	Sensory indicator Yes
Estimated effect (standard error)	0.14 (0.06) *	0	0.34 (0.16) *	0	−0.93 (0.35) **	0	0.44 (0.16) **

Two-sided value of *p*: 0.05 > * > 0.01 > ** > 0.001 > ***. (x) Communication of absent sensory qualities that might affect acceptance on individual level.

## 4. Discussion

In general, the results of the questionnaire revealed an overall positive response for all tested symbols. A broader question is whether this is an indicator, or signal, of the need to introduce alternative ways of communicating about wine (and hence, by extension, other sensorially-complex products). It also raises the question of whether knowledge within the field of crossmodal correspondence could further transfer into the field of consumer communication to optimize consumer-product matching. This study thus aligns with earlier research aiming to improve matching between consumer groups and potential products (Elder and Krishna, 2010; Varela and Ares, 2012; Krishna and Schwarz, 2014; Croijmans and Wang, 2021), but with a different motivation for implementing these strategies.

The respondents showed an overall positive response to the symbols used in the study, especially those focusing on the multisensory experience and indicators focusing on dominant flavors and their dynamic change during the expected palate experience by the consumer. This indicates that symbols may be a consumer-friendly tool in communicating both multisensory changes and more holistic sensory profiles of wines, conveying the overall expected sensory experience of appearance, odor, taste, and tactile sensations. This finding thus answers the call of semiotic studies to further investigate the use of symbols as a potential tool when it comes to communicating sensory attributes and flavor profiles (König and Lick, 2014; van Tonder and Mulder, 2015; Celhay and Remaud, 2018; Pelet et al., 2020). Furthermore, since there was no significant correlation between demographics and flavor intensity, timeline, or the most popular symbols, this type of visual symbolic approach might be a useful crossmodal tool in addressing a broader population by not being a tool for communication to a specific demographic group (Hutmacher, 2019).

A significant majority of the respondents also demonstrated a positive inclination toward seeking evaluations from wine experts to assess aspects like the wine’s readiness, quality, and potential flaws. This inclination underscores the inherent sensory intricacies associated with wine as a product, as well as the persistent desire to enhance communication strategies aimed at bridging the gap between sensory expectations and the ensuing sensory experience. This need for expert assessment sheds light on a possible explanation for the proliferation of websites and mobile applications that prioritize assisting consumers in making informed wine purchases, examples of which include vivino.com, wine-searcher.com, and cellertracker.com.

These online platforms typically offer a diverse array of communication tools designed to support consumers in selecting wine. These tools encompass quality ratings, geographical descriptions, insights into vinification and viticultural practices, purchasing and maturation guidance, and vintage charts. Importantly, they often transcend conventional information parameters such as wine origin, grape variety, producer details, vintage year, and legal specifications. The prevalence and diversity of these online resources may signify a growing recognition of the limitations inherent in traditional approaches to communicating about complex food products such as wine. It’s plausible that the sheer number of these websites and the multifaceted communication tools they provide serve as a testament to the evolving landscape of wine communication, one that seeks to address the nuanced and multifaceted aspects of this sensory-rich domain.

In the present study, respondents’ positive response toward symbols and other visual tools to be applied for crossmodal communication also supports earlier findings, which suggest that linguistic symbolic tools, like metaphors, analogies, metonymies, and allegories, may complement crossmodal communication (Paradis and Eeg-Olofsson, 2013; Moreno Lara, 2014; Alousque, 2015; Kelley et al., 2015; Costello et al., 2018; Herdenstam et al., 2020). Other aspects to consider when developing visual tools for crossmodal communication are which crucial sensory descriptors to select when trying to create olfactory mental images (Tomiczek and Stevenson, 2009) or a more general mental image of the overall sensory experience (Williamson et al., 2016; Croijmans and Wang, 2021; Spence and Van Doorn, 2022; Spence, 2022a).

Other attitudes regarding sensory labeling, blending wines, and sustainability indicate a positive response toward wine with alternative labeling using symbols and strict sensory information. Respondents’ positive response toward testing wine that had been made by blending existing wines, see Wang and Spence (2018a,b), combined with their willingness to make sustainable choices when purchasing, indicate the potential for future wine rescuing programs,. Such programs could use different batches of wines that are left over due to overproduction and/or changes in sensory profile.

Within the respondent group under examination in this study, it was observed that individuals who primarily relied on their prior wine experiences during the purchasing process exhibited reduced interest in communication that pertained to consumer taste group classifications based on genetics. This finding suggests that once consumers have identified a particular style or type of wine that aligns with their preferences, it becomes a potent determinant for their future wine purchases. This influence seems to outweigh the significance of genetic classifications, which can often be challenging to relate to. An alternative explanation could be rooted in the substantial body of research focusing on genetics and preference, which, due to its complexity, may be challenging for consumers to grasp. This complexity arises from the multitude of variables beyond genetics, including environmental factors and cultural influences, which, to a certain extent, necessitate self-awareness, a foundational understanding of genetics, and knowledge of how this genetic information corresponds to their individual sensory experiences (Bartoshuk et al., 1996; Bartoshuk, 2000; Keast and Breslin, 2003; Reed and Knaapila, 2010; Kim et al., 2015; Linscott and Lim, 2016; Herdenstam et al., 2018; Pagliarini et al., 2021).

Conversely, the positive correlations identified between purchasing behaviors and the examined multisensory symbols—comprising the intensity and composition of dominant flavors, as well as the temporal development of pivotal sensory flavors—suggest that these symbols possess potential as crossmodal tools. These tools use visual cues to communicate not only taste, aroma, and tactile sensations, but also the anticipated progression of taste experiences on the palate. This holistic approach aids consumers in grasping the sensory encounter comprehensively. Furthermore, the outcomes of this study could have implications for the context in which the wine is consumed and potential recommendations for certain food pairings that help to enhance the attributes of the wine in the context of the wine-food matching (Harrington, 2005, 2008; Koone et al., 2014; Herdenstam et al., 2018). This study’s focus on vision as a crossmodal tool for communication highlights one part of the multisensory reality that the consumer faces, whether it is the purchasing or the consuming situation or both. The environment in which individuals interact with wine labels is a multisensory, atmospheric, and crossmodal experience on many levels, as has been proposed by earlier studies investigating the multisensory environment (Spence et al., 2014; Spence, 2020b, 2022a).

## 5. Conclusion

Applying alternative labeling approaches with sensory indicators and symbols may better communicate the expected sensory experience in relation to different consumers and their actual preferences. Taken together, accomplishing better communication for food products—in this case wine, which has been refined at many levels, from cultivation, production, maturation, and storage to final distribution to end consumer—also contributes to improved use of natural resources, thus decreasing the climate footprint. Based on the results of the present study, visual crossmodal communication may potentially both grasp critical consumer attributes and convey multisensory experiences, as well as the holistic timeline of taste development. This form of communication may thus be a useful tool in communicating wine and other complex food products.

## 6. Limitations and further research

While the study provides valuable insights into the potential of alternative labeling approaches and visual crossmodal communication for wine and other complex food products, it is important to acknowledge some limitations. The study might have benefited from a larger and more diverse sample. The respondents’ demographics and wine preferences could have been more varied to obtain a broader perspective on the effectiveness of the symbols and visual tools across different consumer groups. The study primarily focused on the visual aspect of crossmodal communication and did not extensively consider other contextual factors that influence wine perception, such as the environment, social context, or individual differences in sensory sensitivity. Future research could explore the interaction between visual symbols and these contextual factors to gain a more comprehensive understanding of crossmodal communication. Investigating the cultural and individual differences in symbol interpretation and understanding would provide valuable insights for effective crossmodal communication.

Future research could address these limitations by conducting larger-scale studies with diverse samples, considering contextual factors, investigating symbol interpretation and design optimization, examining long-term effects, exploring practical implementation challenges, and extending the scope to other sensory-complex products. By addressing these areas, researchers can further advance the understanding and application of crossmodal communication strategies in consumer product matching.

## Data availability statement

The original contributions presented in the study are included in the article/Supplementary material, further inquiries can be directed to the corresponding author.

## Ethics statement

Ethical approval was not required for the study involving humans in accordance with the local legislation and institutional requirements. Written informed consent to participate in this study was not required from the participants or the participants’ legal guardians/next of kin in accordance with the national legislation and the institutional requirements. Written informed consent was obtained from the individual(s) for the publication of any potentially identifiable images or data included in this article.

## Author contributions

AC-F: manuscript and direction. CS: manuscript and direction. MM: philosophical input. NP: statistics and general analysis. All authors contributed to the article and approved the submitted version.

## Conflict of interest

The authors declare that the research was conducted in the absence of any commercial or financial relationships that could be construed as a potential conflict of interest.

## Publisher’s note

All claims expressed in this article are solely those of the authors and do not necessarily represent those of their affiliated organizations, or those of the publisher, the editors and the reviewers. Any product that may be evaluated in this article, or claim that may be made by its manufacturer, is not guaranteed or endorsed by the publisher.
