# Supplementary material for: Enhancing the design of wine labels
Source: Front Psychol. 2023 Sep 25;14:1176794. doi: 10.3389/fpsyg.2023.1176794 (PMC10560737; doi:10.3389/fpsyg.2023.1176794)
Supplement: Supplementary file 1 [file Data_Sheet_1.docx]

APPENDIX A – DEMOGRAPHICS

| **Gender (n=329)** | **Frequency (%)** |  | **Place of consumption (n=329)** | **Frequency (%)** |
| --- | --- | --- | --- | --- |
| Female | 59.3 |  | At home | 80.1 |
| Male | 40.1 |  | At friends or family | 13.2 |
| Other | 0.6 |  | Restaurant | 5.2 |
|  |  |  | Wine tasting group | 0.9 |
| **Where do you live**? (n=329) | Frequency (%) |  | Wine bar | 0.3 |
| Rural community | 14.9 |  | Other | 0.3 |
| Suburbs | 9.1 |  |  |  |
| City | 50.8 |  | **Wine consumption** (n=329) | Frequency (%) |
| Capital city | 25.2 |  | 6 to 7 days a week | 0.9 |
|  |  |  | 4 to 5 days a week | 5.8 |
| **Education** (n=329) | Frequency (%) |  | 2 to 3 days a week | 45.0 |
| Doctorate degree | 4.3 |  | Once a week | 35.3 |
| Master’s degree | 38.0 |  | Once a month | 12.5 |
| Bachelor’s degree | 31.6 |  | Once a year | 0.6 |
| Associate degree | 0.9 |  | Never | 0.0 |
| Trade/Technical/vocational training | 1.5 |  |  |  |
| Some college/university  credits (no diploma) | 19.5 |  | **Purchasing wine** (n=326) | Frequency (%) |
| High school | 4.0 |  | From a monopoly | 81.3 |
| Middle school | 0.3 |  | Liquor/wine store | 7.4 |
|  |  |  | Via a wine importer | 4.9 |
| **Wine Knowledge** (n=329) | Frequency (%) |  | Via a wine producer | 1.2 |
| Experienced, educated | 24.6 |  | Online | 0.0 |
| Experienced, no education | 7.0 |  | At a restaurant or bar | 2.1 |
| Better than average | 44.4 |  | Other | 3.1 |
| Average | 17.6 |  |  |  |
| Poor knowledge, interested | 6.1 |  |  |  |
| Poor knowledge, no interest | 0.3 |  |  |  |
| No knowledge | 0.0 |  |  |  |

APPENDIX B - THE QUESTIONNAIRE
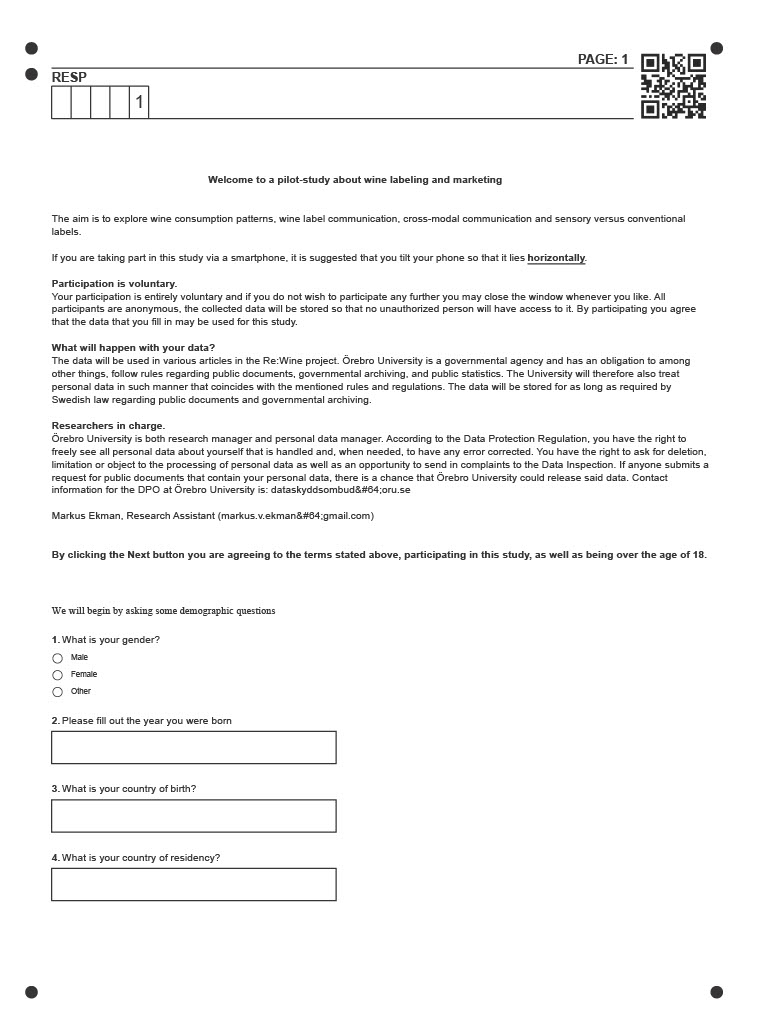

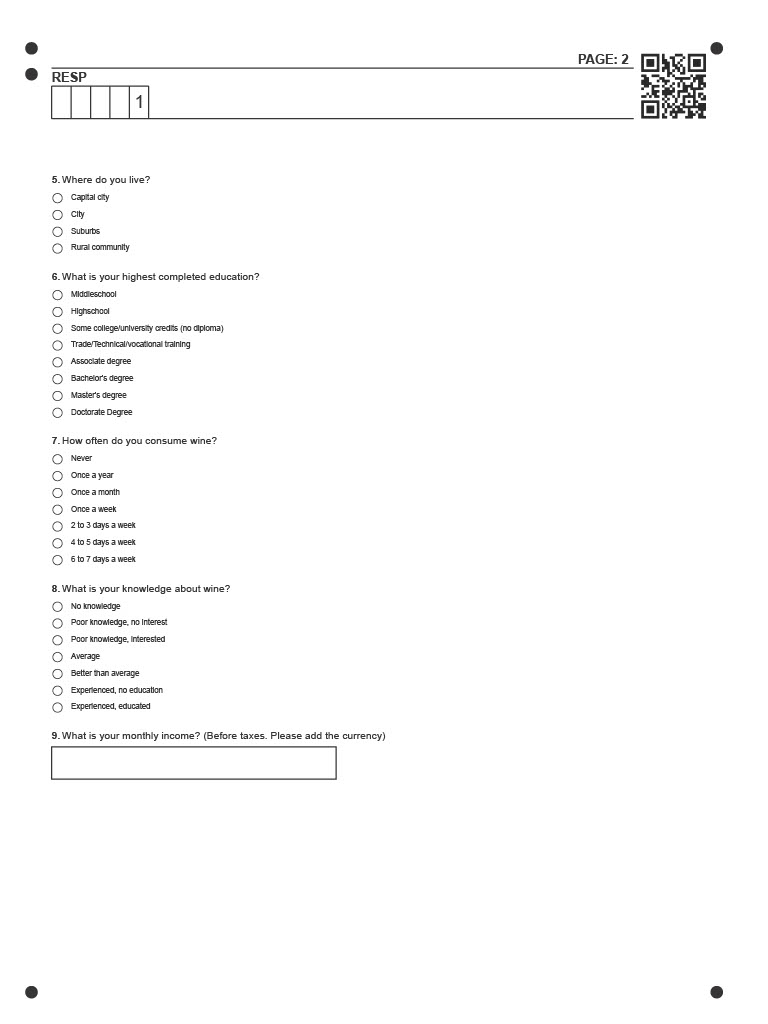

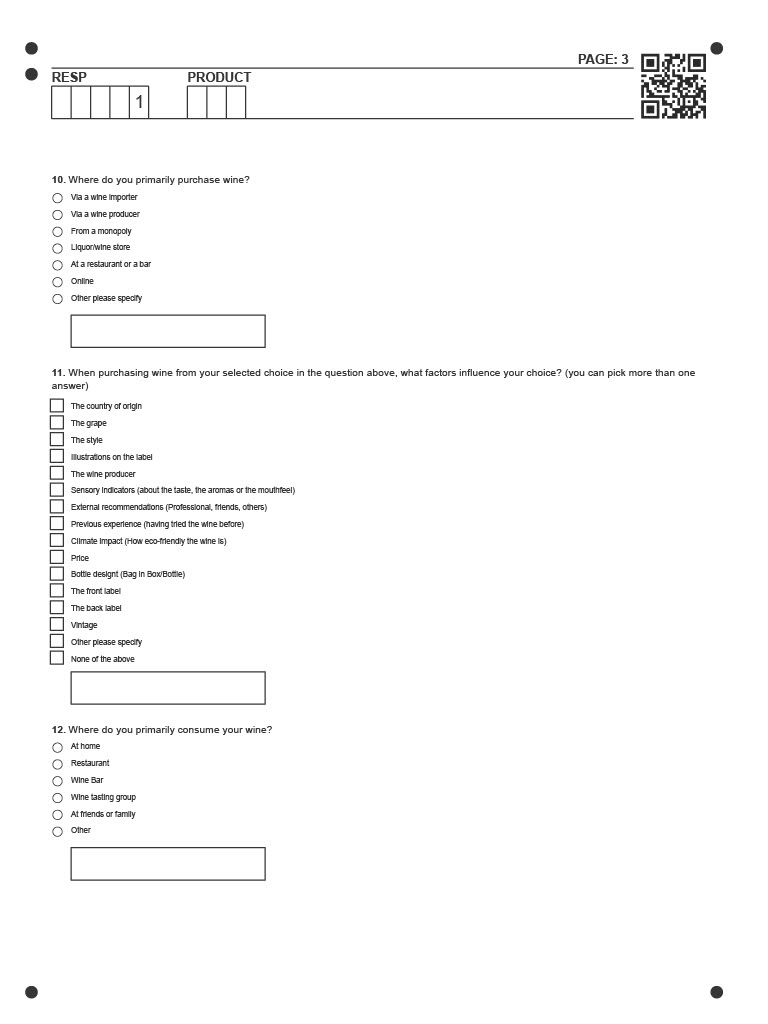

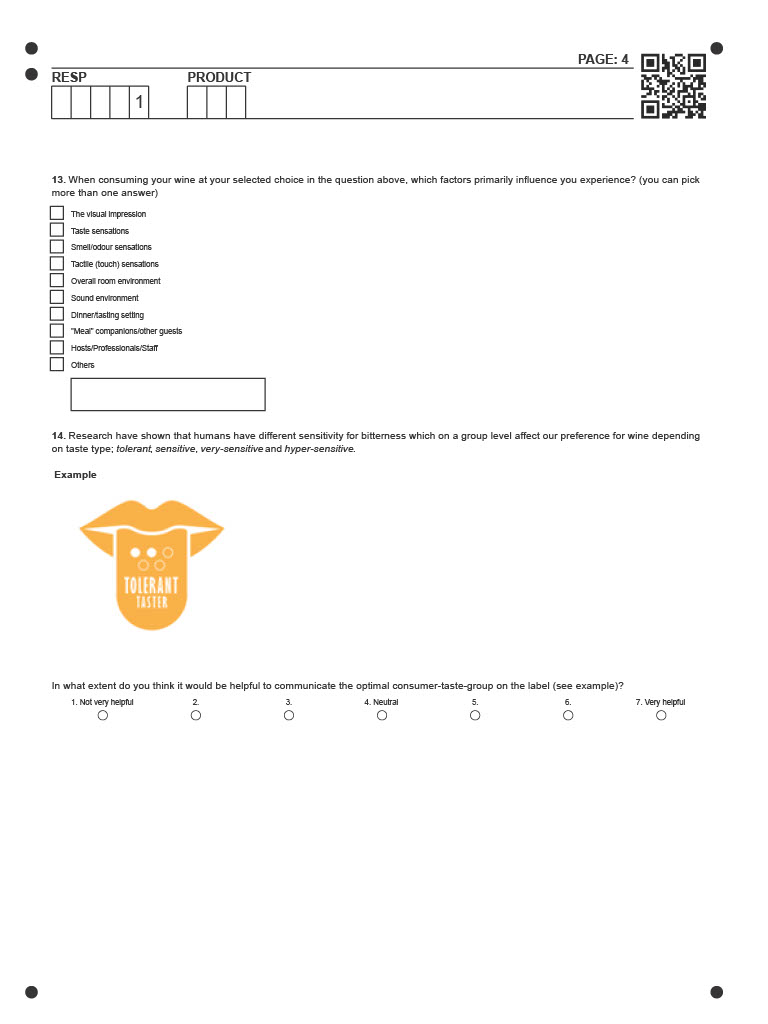

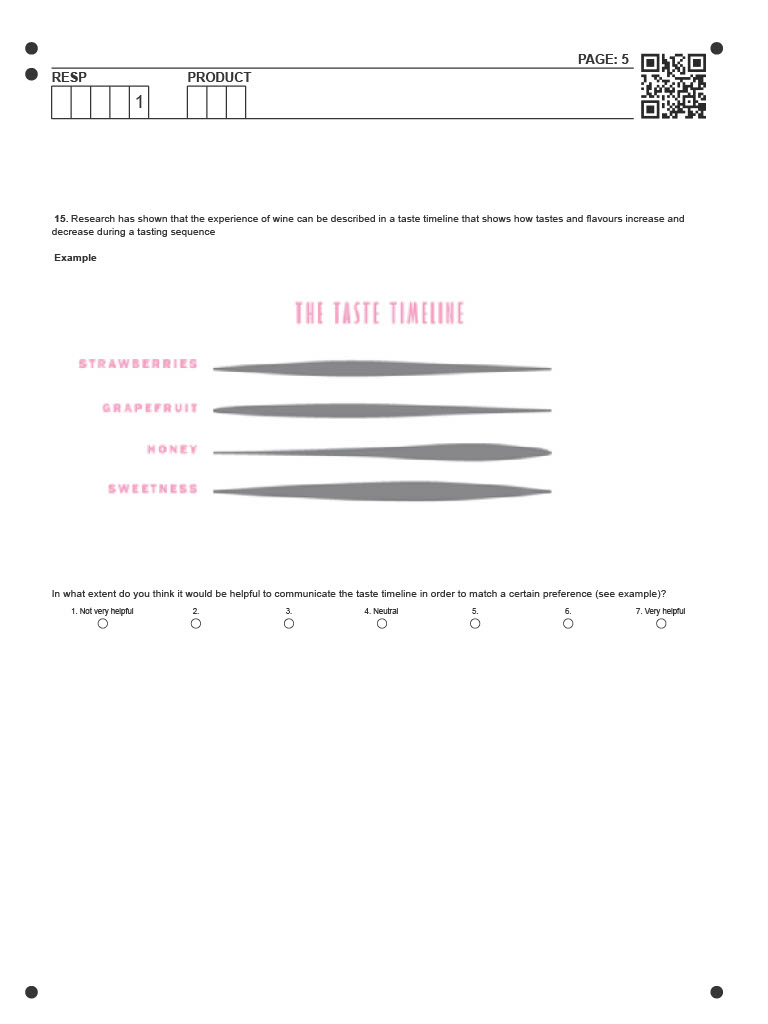

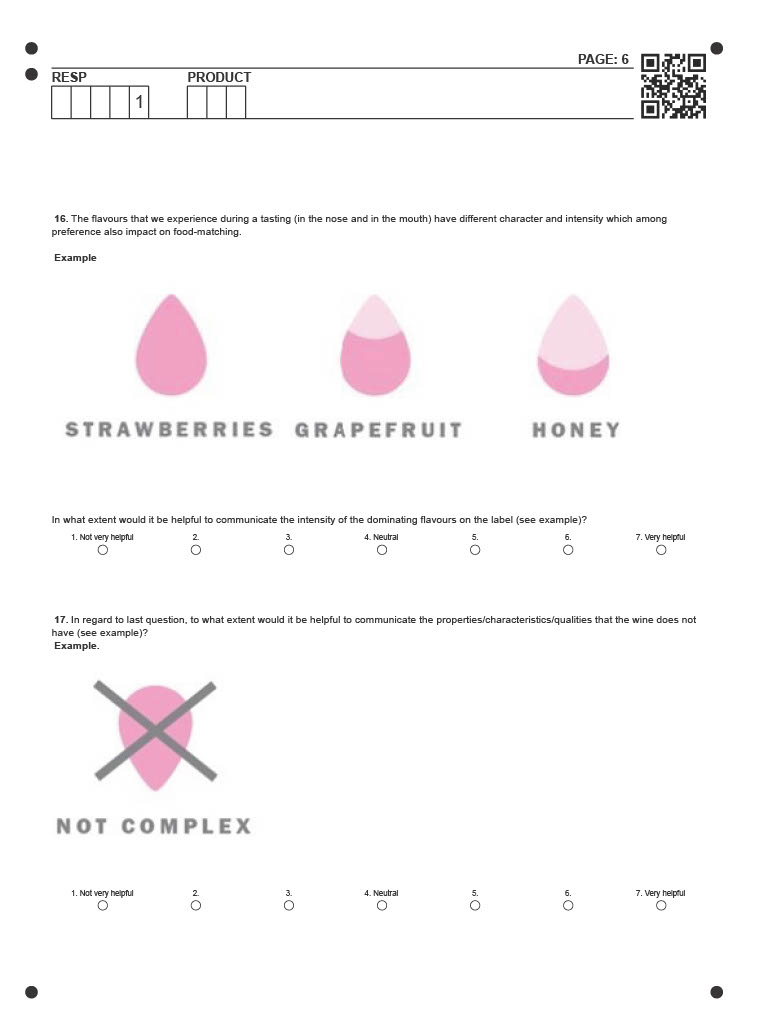

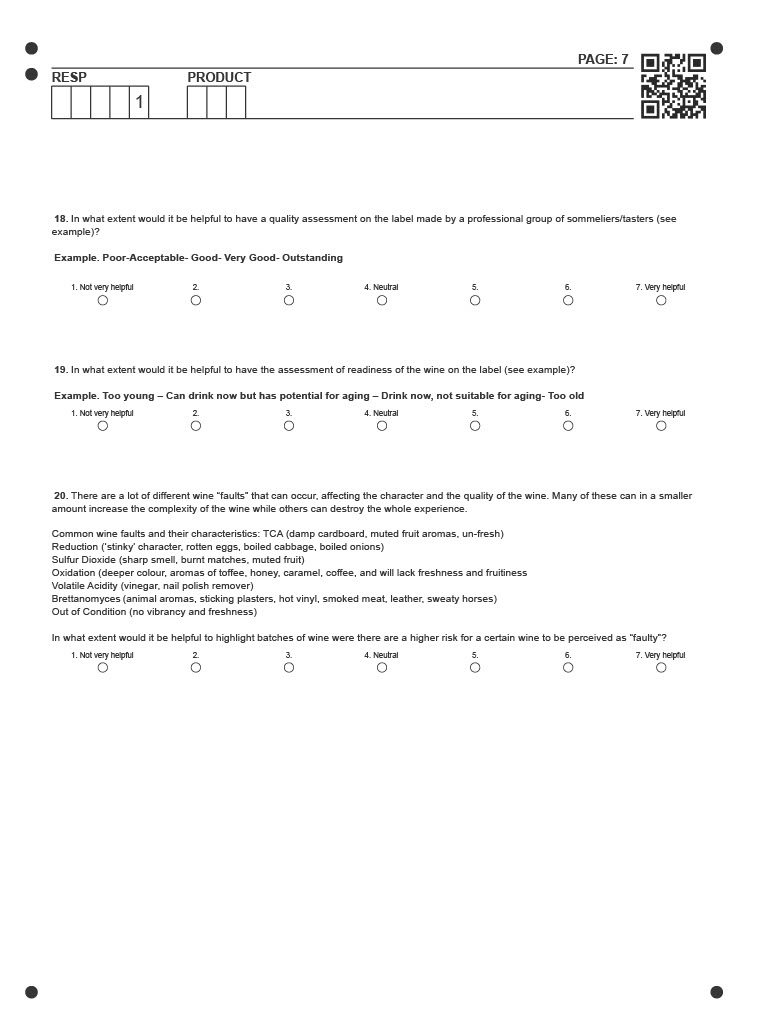

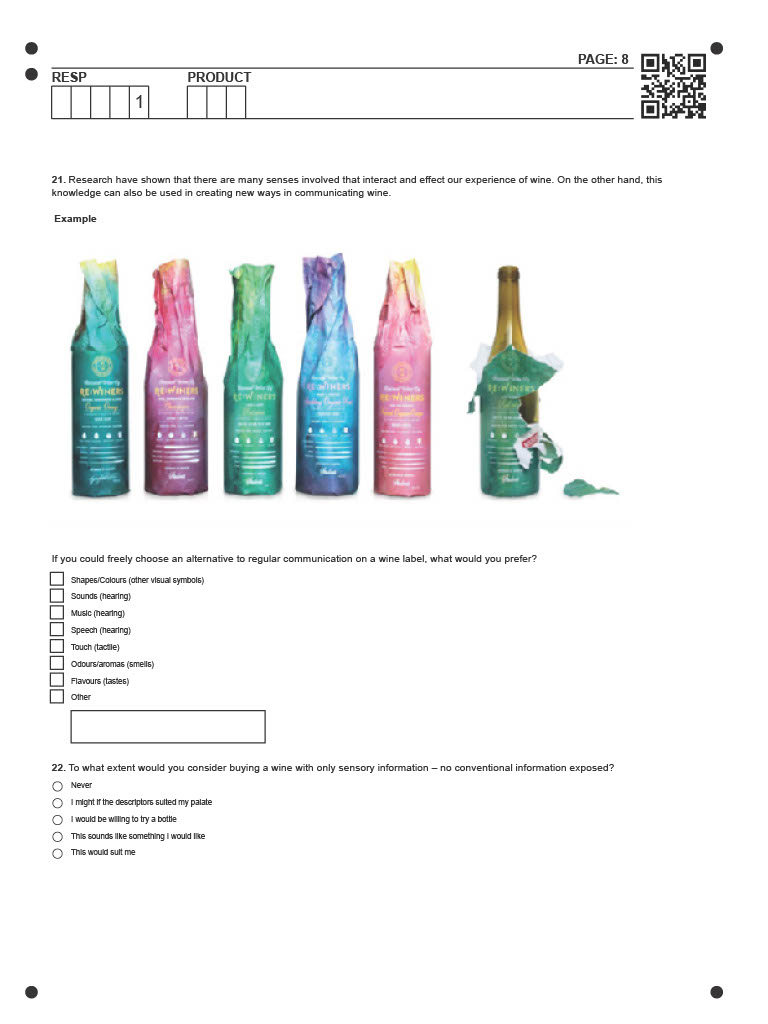

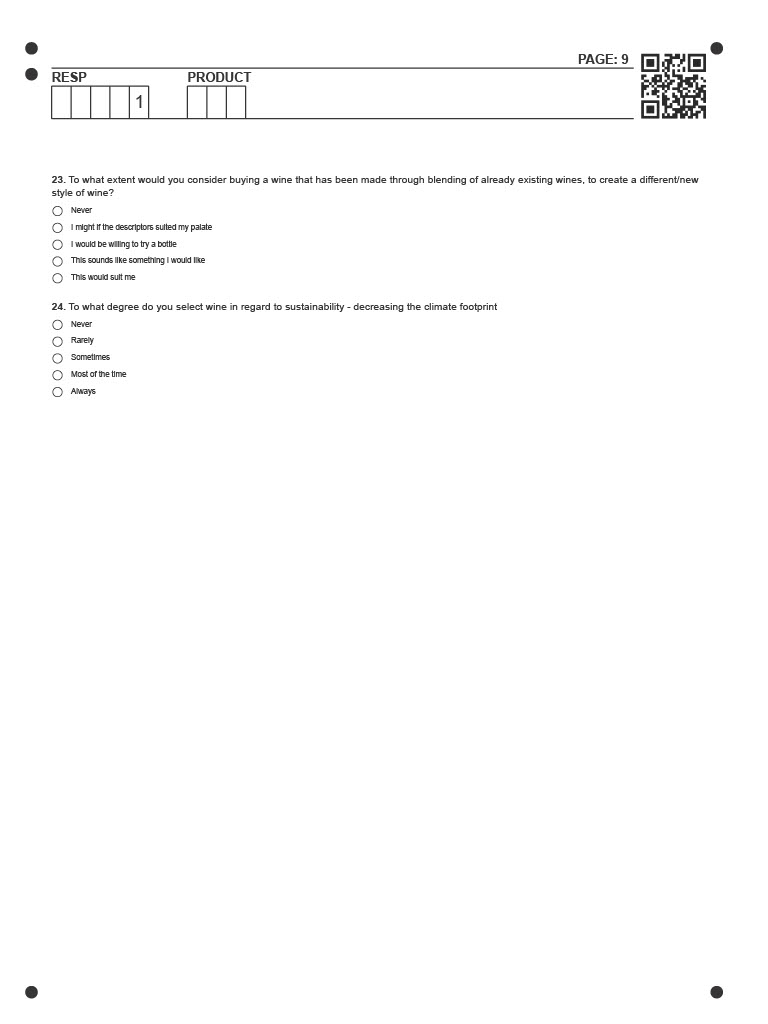

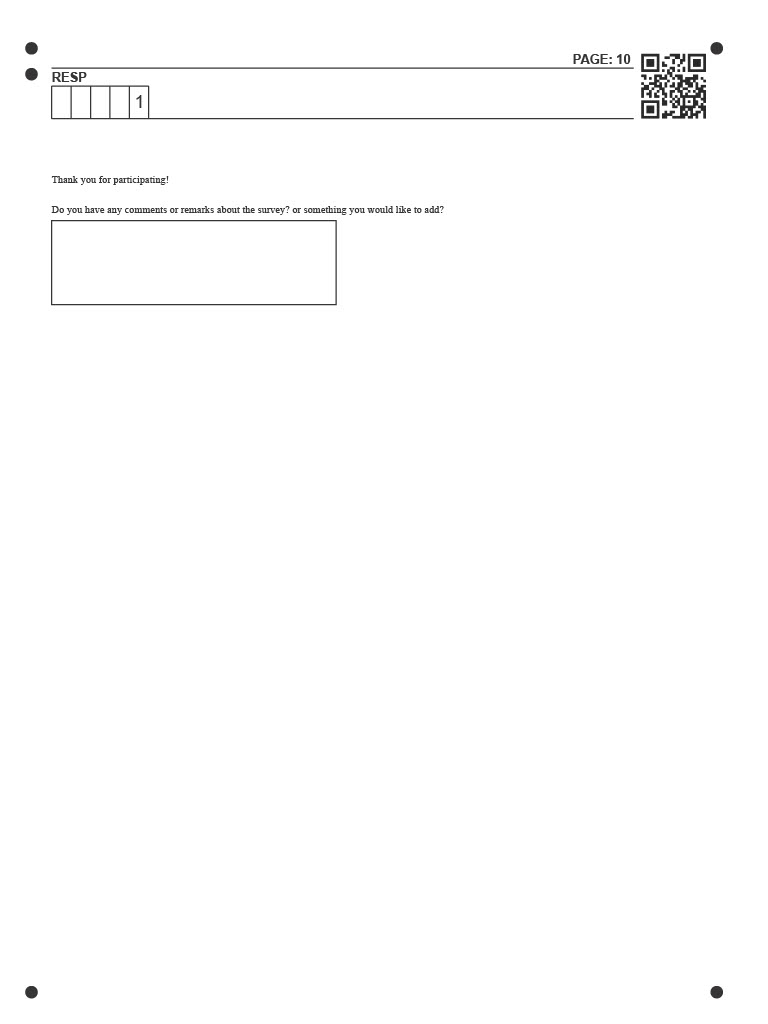


APPENDIX C - Attitudes regarding purchase, consumption, and sensory communication

| **To what extent would you consider buying a wine based only on sensory information – i.e., if no conventional information is presented? (n=315)** | | |
| --- | --- | --- |
| Only sensory information | Frequency (%) | |
| This would suit me | 1.6 | |
| This sounds like something I would like | 15.9 | |
| I would be willing to try a bottle | 46.0 | |
| I might if the descriptors suited my palate | 27.9 | |
| Never | 8.6 | |
|  | |  |
| **To what extent would you consider buying a wine that has been made through blending of already existing wines, to create a different/new style of wine? (n=315)** | | |
| Buying blended wines | Frequency (%) | |
| This would suit me | 3.5 | |
| This sounds like something I would like | 14.9 | |
| I would be willing to try a bottle | 55.2 | |
| I might if the descriptors suited my palate | 21.0 | |
| Never | 5.4 | |
|  | |  |
| **To what extent do you select wine in regard to sustainability - decreasing the climate footprint? (n=315)** | | |
| Sustainability | Frequency (%) | |
| Always | 2.2 | |
| Most of the time | 22.9 | |
| Sometimes | 50.8 | |
| Rarely | 18.4 | |
| Never | 5.7 | |

APPENDIX D Alternative label communication by nominal demographical variables
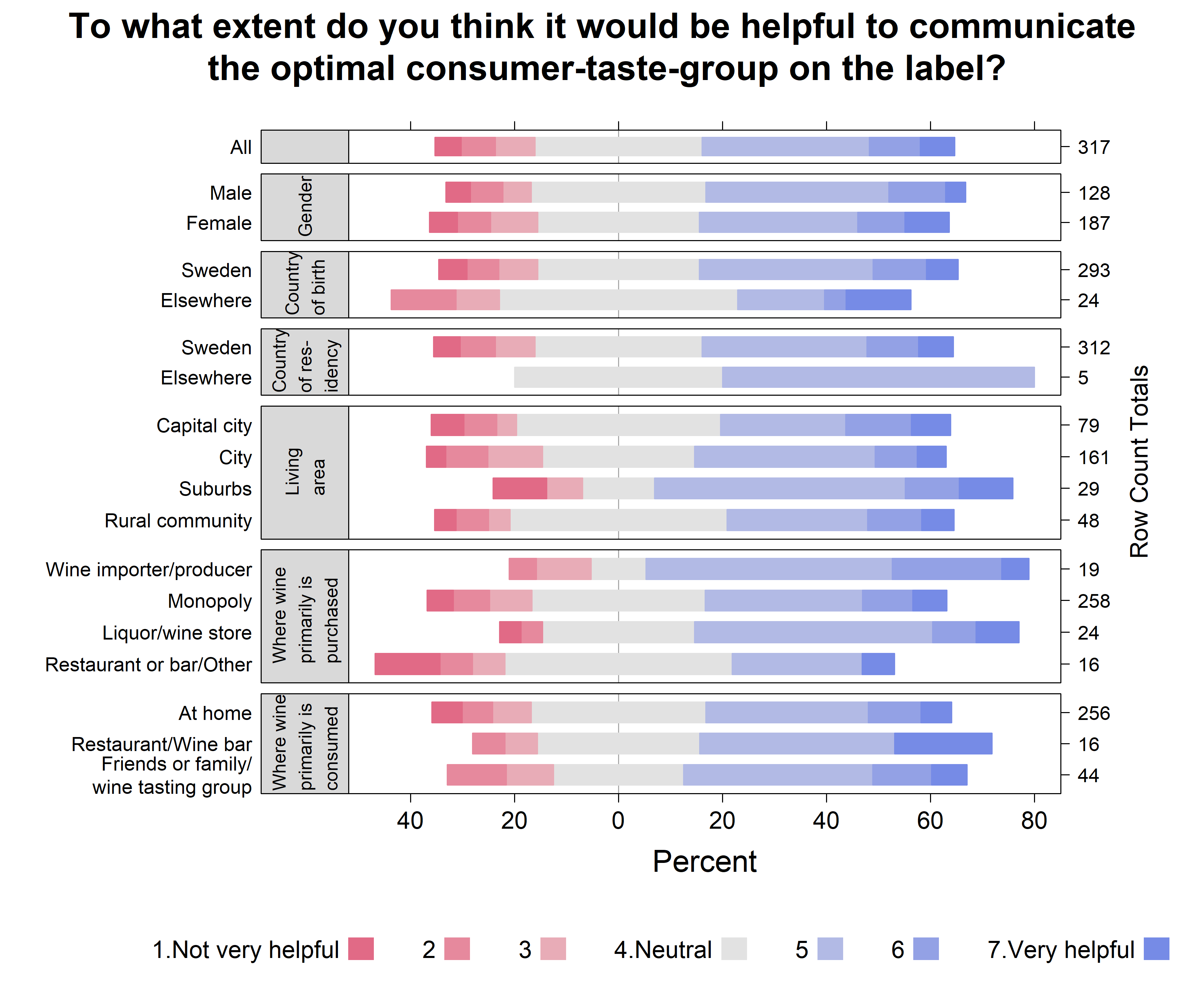


Two-sided hypothesis tests: All had p-value>=0.05.


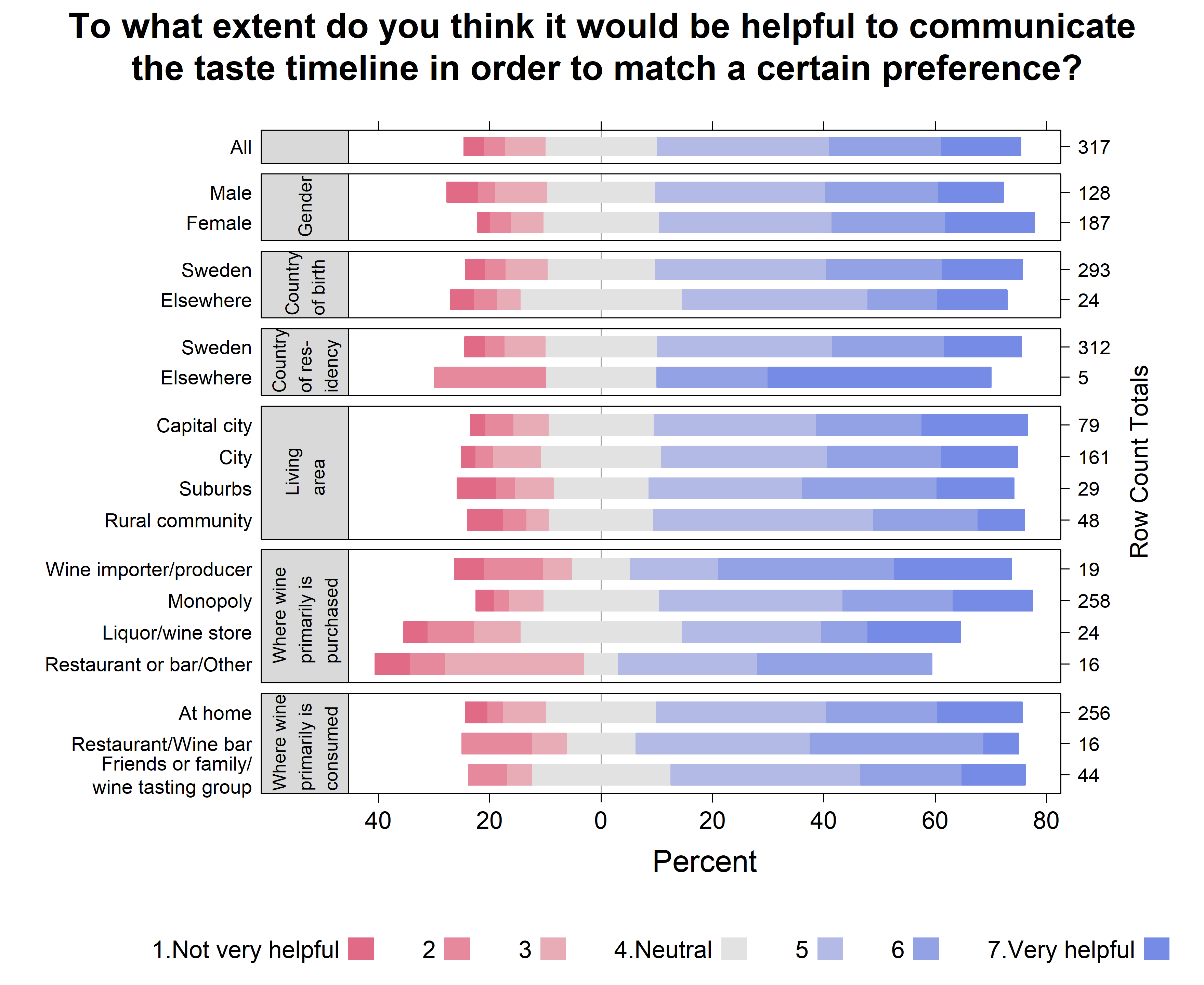


Two-sided hypothesis tests: All had p-value>=0.05.


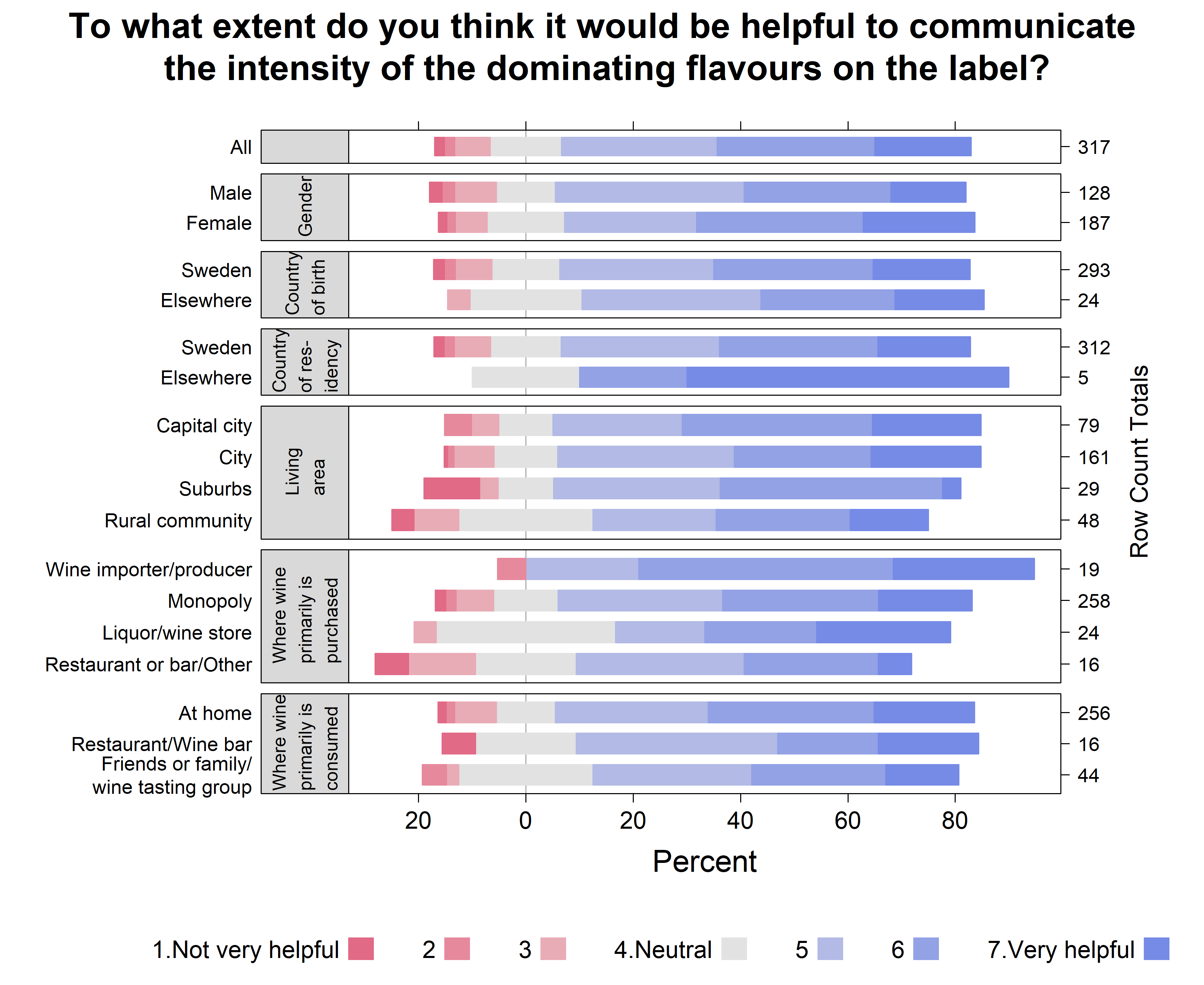


Two-sided hypothesis tests: All had p-value>=0.05.


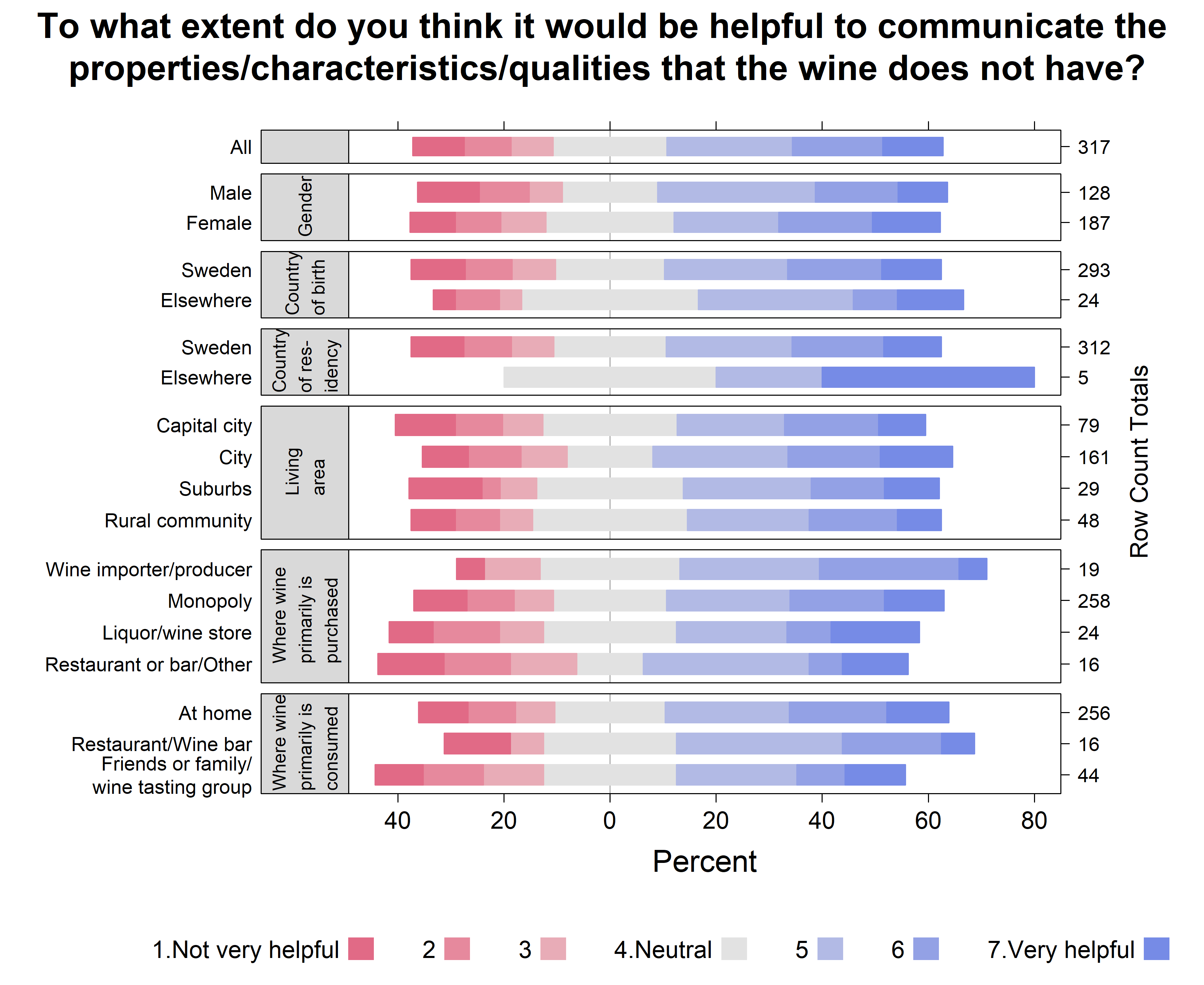


Two-sided hypothesis tests: All had p-value>=0.05.


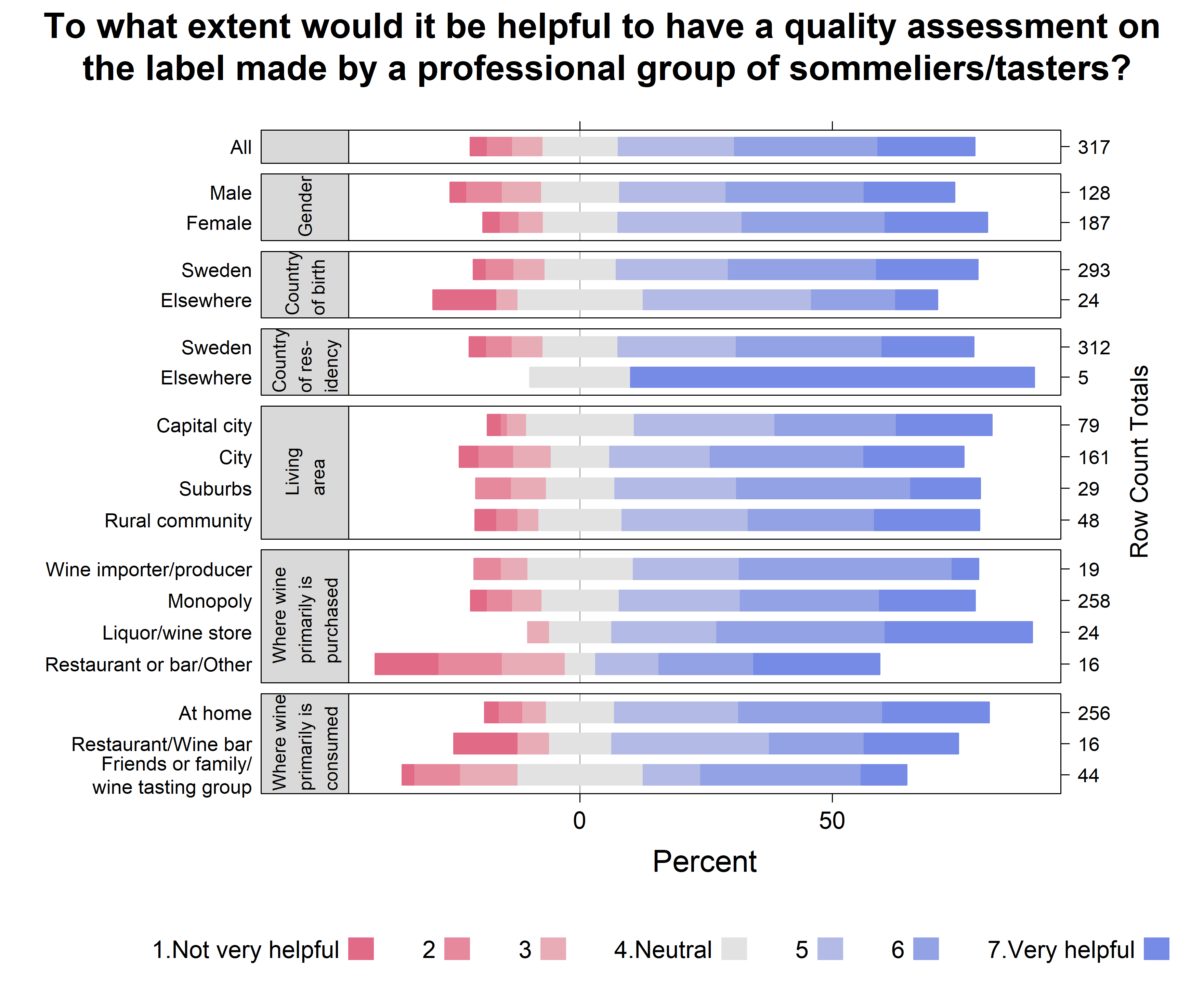


Two-sided hypothesis tests: Country of birth (p-value=0.036). Country of residency (p-value=0.034). Other variables (p-value>=0.05).


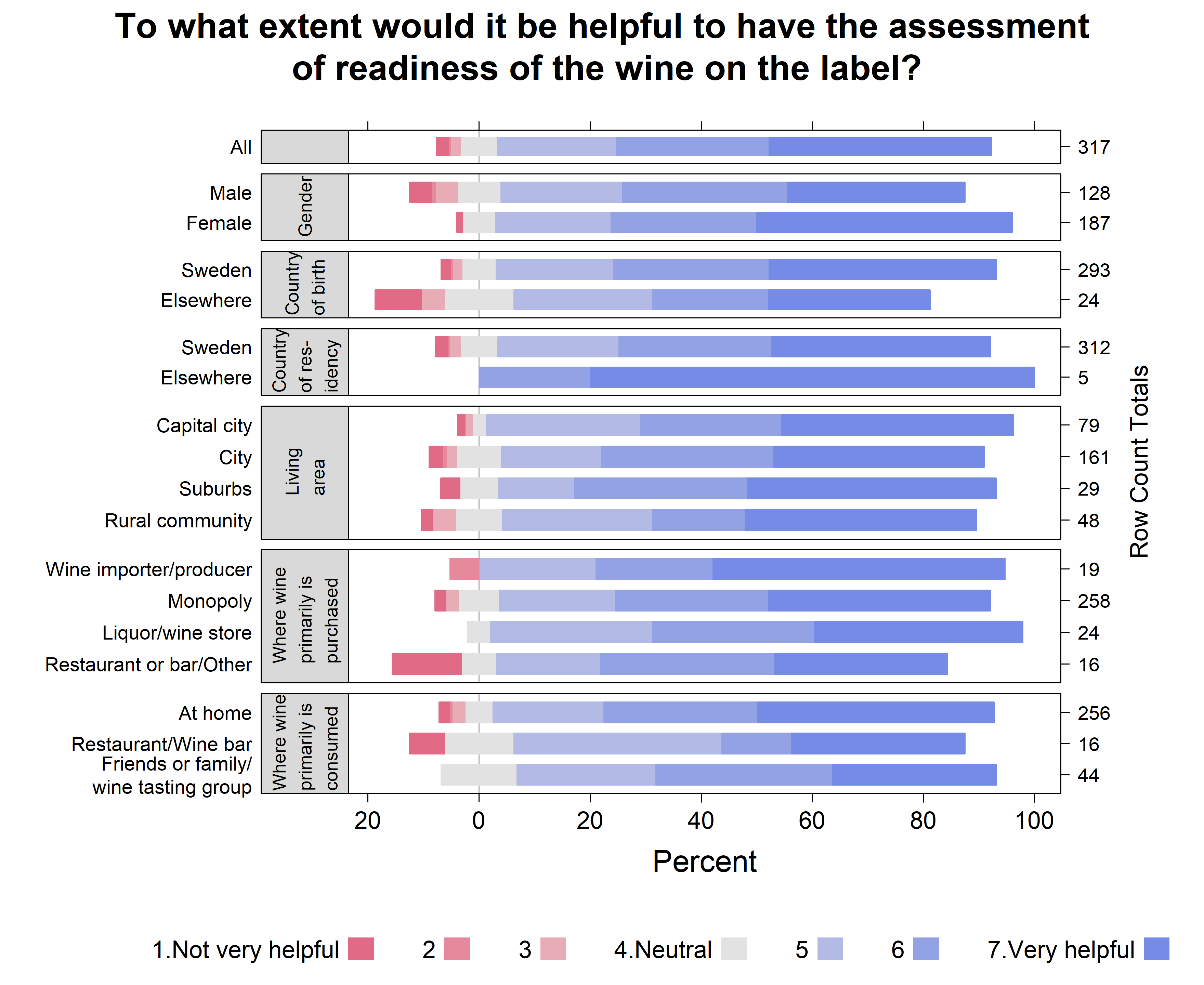


Two-sided hypothesis tests: Gender (p-value=0.004). Other variables (p-value>=0.05).


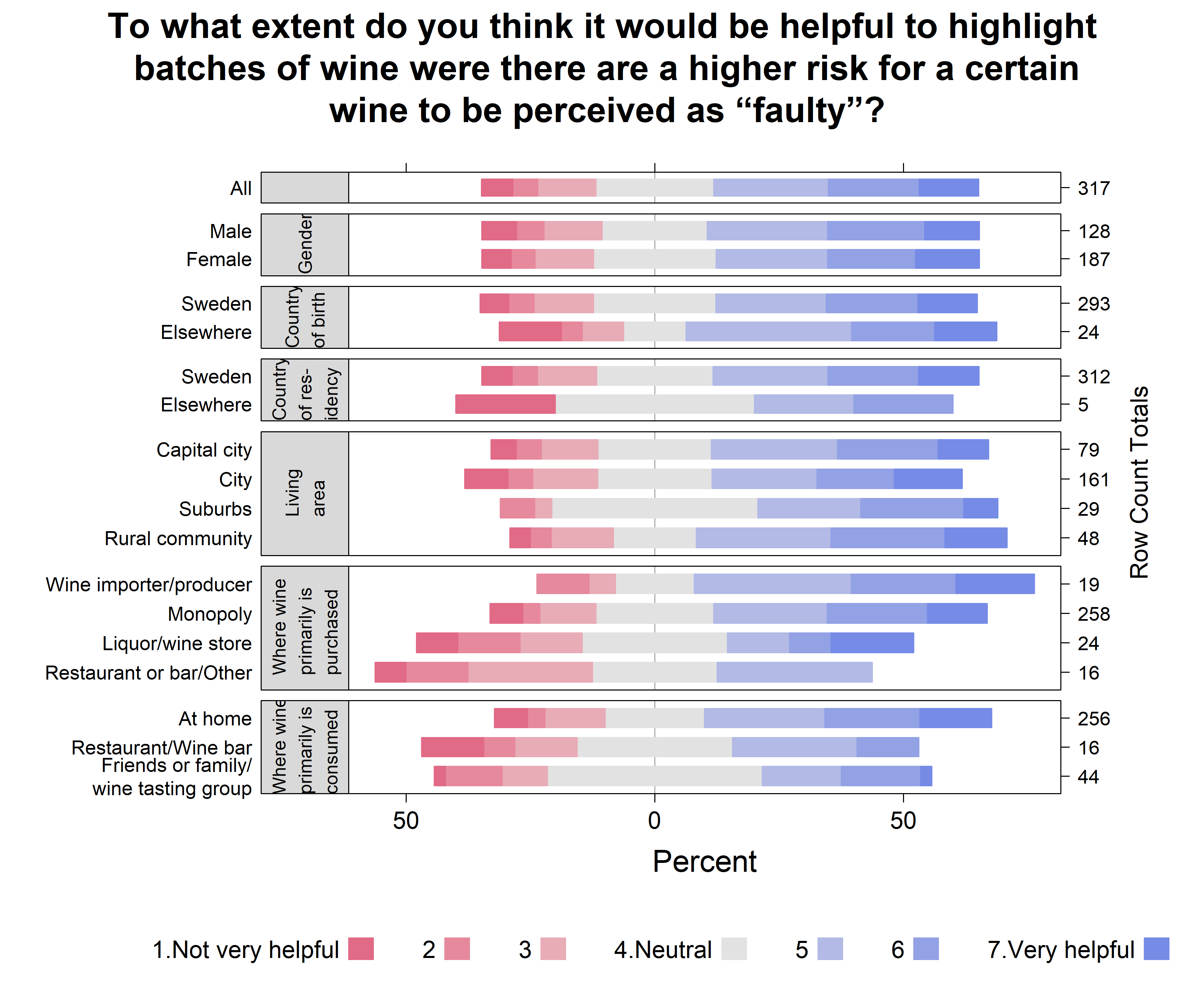


Two-sided hypothesis tests: Where wine primarily is purchased (p-value=0.028). Where wine primarily is consumed (p-value=0.016). Other variables (p-value>=0.05).

APPENDIX E - Correlations (Kendall’s tau) between ‘When consuming your wine at your selected choice in the question above, which factors primarily influence you experience?’ and ‘Perceived helpfulness of alternative label communication’.

|  | **Perceived helpfulness of alternative label communication** | | | | | | |
| --- | --- | --- | --- | --- | --- | --- | --- |
| **When consuming your wine at your selected choice in the question above, which factors primarily influence you experience?** | Genetics | Flavor intensity | Taste timeline | Non-existing qualities (x) | Quality by pro | Readiness (to drink) | High-risk faults |
| Visual impression | 0.009 | 0.040 | 0.095 | 0.093 | -0.005 | 0.038 | -0.008 |
| Taste sensations | 0.030 | -0.033 | 0.012 | -0.054 | -0.053 | 0.061 | -0.001 |
| Smell/odor sensations | -0.009 | 0.035 | 0.031 | -0.026 | -0.037 | 0.045 | 0.014 |
| Tactile sensations | 0.012 | 0.090 | -0.003 | 0.059 | -0.039 | -0.058 | -0.013 |
| Overall room environment | 0.035 | -0.041 | -0.014 | -0.031 | -0.082 | 0.020 | -0.033 |
| Sound environment | 0.006 | -0.038 | -0.027 | -0.019 | -0.092 | -0.029 | -0.004 |
| Dinner/tasting setting | -0.072 | -0.090 | 0.066 | -0.043 | 0.056 | 0.004 | -0.024 |
| Hosts/Professionals/Staff | -0.022 | -0.047 | 0.011 | -0.001 | 0.023 | 0.060 | -0.043 |
| Others | -0.033 | -0.037 | 0.015 | 0.058 | -0.000 | 0.003 | -0.045 |

Two-sided p-value: 0.05 > * > 0.01 > ** > 0.001 > ***.

(x) Communication of absent sensory qualities that might affect acceptance on individual level.

APPENDIX F -Wines and other beverages tasted during the course

| **Theme - Wine Tasting Methodology** | **Volume (ml)** | **Price (SEK)** |
| --- | --- | --- |
| Moncaro Trebbiano | 750 | 59 |
| Gustave Lorentz Gewurztraminer Réserve | 750 | 109 |
| Lindemans Bin 65 Chardonnay | 375 | 47 |
| Valpolicella Bonacosta, Masi | 750 | 119 |
| Barolo Serralunga d’Alba, Fontanafredda | 375 | 169 |
| Riesling Beerenauslese, Dr Loosen | 187 | 129 |
|  |  |  |
| **Theme - Quality** |  |  |
| Orballo Albariño | 750 | 117 |
| Allesverloren Chenin Blanc | 750 | 105 |
| Errazuriz Cabernet Sauvignon Rosé, | 375 | 49 |
| Barefoot White Zinfandel | 250 | 34 |
| Château de Pierreux, Beaujolais | 750 | 129 |
| Bonterra Zinfandel | 750 | 159 |
|  |  |  |
| **Theme - Grapes** |  |  |
| Riesling: Leitz Magic Mountain Rüdesheimer Trocken (750 ml), Tyskland, | 750 | 159 |
| Sauvignon Blanc: Sancerre Domaine des Vieux Pruniers, Loire | 375 | 109 |
| Chardonnay: Bourgogne Les Murelles Domaine Roux | 375 | 85 |
| Pinot Noir: Bourgogne Cuvée Margot, Olivier Leflaive | 750 | 219 |
| Syrah: Delas Les Launes Crozes-Hermitage | 375 | 89 |
| Cabernet Sauvignon/Merlot: Château la Tour de By | 375 | 89 |
|  |  |  |
| **Theme – Wines in the World** |  |  |
| Langhe Nebbiolo Paolo Scavino | 375 | 99 |
| Marqués de Arienzo Reserva, Rioja | 375 | 79 |
| Portuga Reserva, nr 2507, 59 kronor | 750 | 59 |
| Penfolds Kalimna Bin 28 Shiraz | 750 | 249 |
| Fleur du Cap Essence du Cap Pinotage, | 375 | 49 |
| Seghesio Sonoma Zinfandel | 750 | 223 |
| Navarro Correas Reserve Malbec | 375 | 59 |
|  |  |  |
| **Theme - Sparkling** |  |  |
| Chapel Hill Sparkling Chardonnay | 200 | 29 |
| Prosecco Pizzolato | 200 | 42 |
| Castiôn Moscato d'Asti | 750 | 95 |
| Pongrácz Brut | 750 | 120 |
| MIM Natura Brut Nature Reserva | 750 | 134 |
| Vve Fourny & Fils Blanc de Blancs Brut Premier Cru | 750 | 329 |
|  |  |  |
| **Theme - Spirits** |  |  |
| Gin: Beefeater London Dry Gin, | 350 | 140 |
| Cognac: Martell VS Single Distillery | 350 | 199 |
| Whisky: Laphroaig 10 Years Old | 350 | 269 |
|  |  |  |
| **Theme – Sweet wines and Fortified** |  |  |
| Castelnau de Suduiraut | 375 | 169 |
| Delas La Pastourelle Muscat de Beaumes de Venise | 375 | 89 |
| Tio Pepe Fino | 375 | 64 |
| Lustau Solera Reserva Amontillado Los Arcos | 375 | 104 |
| Churchill’s Late Bottled Vintage | 375 | 109 |
| J.H. Goncalves Sercial 5 Years | 375 | 109 |
|  |  |  |
| **Theme – Beer** |  |  |
| Nynäshamns Landsort Lager, Sweden | 500 | 29 |
| Staropramen Dark, Czech Republic | 330 | 18 |
| Oppigårds Indian Tribute, Sweden | 330 | 24 |
| Yeti Imperial Stout, USA | 355 | 42 |
| Weihenstephaner Hefe Weissbier, Germany | 500 | 24 |
| Oude Kriek Boon, Belgium | 375 | 60 |
|  |  |  |
| **Theme - Cider** |  |  |
| Cidre Biologique Brut, Normandie | 750 | 54 |
| Gaymer's Olde English Cyder | 1000 | 50 |
| Petritegi Sidra Natural | 750 | 100 |
|  |  |  |
| **Theme - Non-Alcoholic** |  |  |
| Nigrum, Sweden | 375 | 42 |
| Mikkeller Drink'in The Sun, International | 330 | 20 |
| Domaine de la Prade Organic Merlot Shiraz | 750 | 89 |
|  |  |  |
| **Examination** |  |  |
| Saint Clair Pioneer Block | 750 | 129 |
| Garzón Reserva Tannat | 750 | 129 |
